# When and How Can Endpoints Be Changed after Initiation of a Randomized Clinical Trial?

**DOI:** 10.1371/journal.pctr.0020018

**Published:** 2007-04-13

**Authors:** Scott Evans

## Introduction

Endpoints are outcome measures used to address the objectives of a clinical trial. The primary endpoint is the most important outcome and is used to assess the primary objective of a trial (e.g., the variable used to compare the effect difference of two treatment groups). A fundamental principle in the design of randomized trials involves setting out in advance the endpoints that will be assessed in the trial [1], as failure to prespecify endpoints can introduce bias into a trial and creates opportunities for manipulation. However, sometimes new information may come to light that could merit changes to endpoints during the course of a trial. This new information might include, for example, results from other trials or identification of better biomarkers or surrogate outcome measures. Such changes can allow incorporation of up-to-date knowledge into the trial design. However, changes to endpoints can also compromise the scientific integrity of a trial. Here I discuss some of the issues and decision-making processes that should be considered when evaluating whether to make changes to endpoints, and discuss the documentation and reporting of clinical trials that have revised endpoints.

## Changing Endpoints

Many trials have changed their study endpoints after trial initiation. For example, a recent study [2] concluded that pioglitazone was associated with a significantly longer period in which patients remained free from death, myocardial infarction, or stroke, which was a composite endpoint. Conclusions from this report were questioned [3] because it was believed that the composite endpoint was not prespecified. This belief was based upon a previous publication [4] which listed the trial endpoints, but did not identify this specific composite. Authors of the original article responded to this criticism [5], stating that after initiation of the trial, the study executive committee recognized that the composite endpoint was not part of the original statistical analysis plan and thus the composite was subsequently added. The trialists also noted that the composite endpoint was documented in a revised analysis plan before unblinding the trial data.

More generally, Chan et al. [6] compared published articles with protocols for 102 randomized trials approved by the Scientific-Ethical Committees for Copenhagen and Frederiksberg, Denmark in 1994–1995, and reported that 62% of the trials had at least one primary endpoint that had been changed, introduced, or omitted. Chan et al. [7] compared published articles with protocols for 48 randomized trials approved for funding by the Canadian Institutes of Health Research in 1990–1998, and reported that primary endpoints differed between protocols and publications in 40% of the trials. Given that changes to endpoints are so frequent, it's important to evaluate when such changes are appropriate and how they should be reported.

## Guiding Principles

The principle consideration when evaluating whether to modify an endpoint is whether the decision is independent of the data obtained from the trial to date. If the decision to revise endpoints is independent of the data from the trial, then such revisions may have merit. In fact, Wittes [8] encourages consideration of changes in long-term trials, as medical knowledge evolves or when assumptions made in design of the trial appear questionable. Wittes further argues that researchers “may consider changes to the primary endpoint when the trial has airtight procedures to guarantee separation of the people involved in making such changes from data that could provide insight into treatment effect” [8].

Some trials have successfully changed endpoints after the trials began by maintaining independence between the decision and the trial data. For example, the randomized Post-CABG (Post Coronary Artery Bypass Graft) trial [9] compared two lipid-lowering regimens in patients who had coronary artery bypass surgery. The investigators explicitly did not identify a primary endpoint when they designed the trial. An angiogram to assess lipid deposition in the coronary arteries was conducted at entry and then again five years later. The researchers planned to compare changes in lipid deposition over the five-year interval between the two regimens. Because by design no endpoint would be available for five years after randomizing the first participant, the protocol team used this period to define the endpoint and to develop methods for analyses. Although the endpoint was not prespecified in the design phase, a practice that is not generally recommended, trial leadership ensured that the selection of the endpoint was independent of data from the trial.

**Box 1. Proposal for Handling Changes in Endpoints in Clinical Trials**

**Questions to Ask:**

- What is the source of the new information that triggers consideration of a change in endpoints?
- Have interim data on the endpoint (or related data) been reviewed?
- Who is making the decision to change endpoints? Are trial sponsors involved, or is there an independent external advisory committee?

**Documenting the Endpoint Changes:**

- Update the protocol in a formal protocol amendment.
- Update the clinical trial registry record.
- Revise the statistical analysis plan.

**Reporting the Trial Results:**

- Include a clear statement describing the changes in endpoints, and the information obtained after the start of the trial that led to these changes.
- Include a description of the reasons for these changes and the decision-making procedure.
- Consider the potential biases that may have come about as a result of the change in endpoints.
- Consider including a disclaimer that the results should be interpreted with caution and may need to be confirmed in future trials.
- Report the reasons for excluding endpoints from the analyses and whether this was independent of trial data.

If, however, the decision to change the endpoint is not independent of the trial data, then “cherry-picking” is a serious concern. New endpoints may be selected because they displayed a trend towards significance, while other candidate endpoints may have been examined but not selected or reported because they failed to display a desirable trend; this increases the chance of false positive (type 1) errors. In the Physicians' Health Study [10,11], the trial's data monitoring committee (DMC) recommended termination of the study because interim data seemed unlikely to show any benefit of aspirin with respect to the primary endpoint, total mortality. At the time this decision was made, there was evidence of benefit with respect to myocardial infarction. However, the United States Food and Drug Administration did not approve an indication for aspirin for the prevention of myocardial infarction, because this was not the prespecified primary endpoint.

## When Is a Decision Independent of Data?

To evaluate whether a change in endpoint is independent of data from the trial, investigators and reviewers should ask three important questions. First, what is the source of the new information that elicits consideration of the change in endpoints? If the source is external to the trial in question, for example arising from results from another trial, then the revision of endpoints may be credible. Second, have interim data on the endpoint (or related data) from a trial been reviewed? If trial data have not been reviewed, then the revision of endpoints may again be credible. Third, and most importantly, who is making the decision regarding endpoint revision (e.g., trial sponsors or an independent external advisory committee)? Appropriate decision makers should have no knowledge of the endpoint (or related trial data) results. In particular, if interim analyses have been conducted, the decision makers should not have knowledge of those data. Note, however, that even if no formal interim analysis has been conducted, any impressions that the investigators may have of the trial to date may influence decisions regarding changes in endpoints. For example, investigators may have a “sense” of the endpoint result or a related variable even though formal analysis of the endpoint has not been conducted. An investigator may notice changes in certain patients at his or her site and may attribute these changes to the investigational medication. This can be particularly problematic in unblinded trials. For these reasons, study sponsors, investigators, and DMCs may not be appropriate decision makers for endpoint revisions.

## Appropriate Decision Makers

Since the decision to revise endpoints should be independent of the trial data, a DMC that has reviewed interim data may not be appropriate for making decisions regarding endpoint revisions. Even DMC review of pooled data can suggest treatment effects (e.g., in a two-group comparison study of response rates, a very high pooled response implies a relatively high response rate in both groups). In this case, trial leadership may wish to convene an external advisory committee that has not reviewed data from the trial to assess the potential impact on the integrity of the trial and to make recommendations regarding endpoint revision.

## Scientific Relevance

It is also important to consider the scientific relevance of the endpoints in question. Does the current state of knowledge make the results of the current trial uninformative or inefficient? Is the trial now scientifically uninteresting or irrelevant? If so, then changing endpoints may be constructive, and perhaps even ethically necessary, to ensure that the study generates a scientific contribution. For example, new scientific questions may arise after recently completed trials have already answered the original question of interest. Also, better biomarkers or surrogates may have been identified, or there may have been changes in regulatory oversight.

One should be cautious of potential operational bias induced by the revision of endpoints. Operational bias is created when the conduct of clinical investigators or participants is changed by knowledge (or perceived knowledge) of trial data. Knowledge of revisions to endpoints may influence the actions of clinical investigators or participants as they anticipate the reasons for such revisions. For example, if a decision to change the primary endpoint is made, then participating clinicians and patients may believe that such a change was made due to a lack of efficacy of the intervention. This belief may affect their willingness to participate, affecting accrual and retention.

## Documentation and Reporting

If the trial leadership decides to modify endpoints, then appropriate documentation is crucial. Changes should be described in amendments to the protocol and the analysis plan. The registry record for the trial should also be updated.

Changes in endpoints should also be declared when submitting a manuscript to a journal, so that the results can be properly evaluated. Reporting of a clinical trial with any modified endpoint should include: (1) a clear statement describing the fact that information obtained after trial initiation led to the change in endpoint; (2) a description of the reasons (e.g., whether the endpoint was suggested by the data) and decision procedure (e.g., who made the decision and whether data were unblinded); (3) a discussion of the potential biases induced by the change of the endpoints; (4) if warranted, (i.e., if the decision to add endpoints was not independent of the data), a disclaimer that the results should be interpreted with caution and should be confirmed in future trials; and (5) a report of the reasons for excluding endpoints from the analyses and whether this was independent of trial data. Addressing these items will help ensure clarity and transparency of the analyses, enable the evaluation of the independence of the endpoint revision and trial data as well as the potential for selective reporting, allow assessment of the ramifications of the endpoint revision, and help avoid overinterpretation of the data. Researchers may further consider focusing on descriptive analyses using confidence intervals rather than hypothesis testing to avoid overstating the significance of the results.

Hawkey [12] suggests that journals require submission of the protocol alongside manuscripts describing clinical trial results, to help ensure that the reported endpoints indeed reflect what was defined at the start of the trial. Several journals have adopted this policy, including *PLoS Clinical Trials, The Lancet,* and the *British Medical Journal.* Other journals are considering a requirement to submit raw data (see the Harvard School of Public Health's Workshop on Assuring the Integrity of Reporting and Patient Safety in Therapeutic Trials; http://www.biostat.harvard.edu/events/schering-plough/agenda.html). Notably, for industry-sponsored studies, the *Journal of the American Medical Association* is requiring that analyses be conducted by an independent statistician at an academic institution, in part to protect against post hoc endpoint revisions.

## Precise Definitions

Often, prespecified endpoints are defined vaguely or ambiguously. For example, a protocol designed to study the effects of 24 weeks of a new investigational drug on immune function might specify “CD4 count” as an endpoint. This endpoint could be interpreted in many different ways, including, for example: (1) CD4 count at week 24; (2) changes from baseline in CD4 count at week 24; (3) the occurrence of a doubling of CD4 count from baseline; or (4) the occurrence of at least a 50-cell increase in CD4 count from baseline. If a precise definition and analysis for each endpoint are not specified in advance, it is possible for many different versions of the endpoint to be examined, followed by selection and reporting of the most desirable result. This form of “cherry-picking” inflates the false positive error rate and leads to an underreporting of negative evidence. Thus it is critical to prespecify the precise definition of the primary endpoint together with the method of statistical analysis that will be applied [1].

## An Alternative for Large Trials

In certain cases, it may be appropriate to change or identify endpoints after initiation of a trial, even when the decision is based on data from the trial. For example, if a trial is very large and of long duration, then investigators may divide the trial into two stages: a hypothesis-generating stage in which endpoints are identified, and a subsequent hypothesis-testing stage. In this case, statistical testing would be based only on data collected after the first stage was complete.

## Conclusions

Revisions to endpoints (particularly primary endpoints) should be uncommon. If not appropriately evaluated, such revisions lead to misguided research and suboptimal patient care. If, however, important scientific knowledge has been gained after a trial begins, then this knowledge should be carefully and responsibly evaluated for incorporation into the trial. We should be open-minded and flexible in situations that may warrant the revision of endpoints and apply appropriate decision-making and reporting procedures when such situations arise.

## Abbreviations

DMC: data monitoring committee
